# Rapid preparation and antimicrobial activity of polyurea coatings with RE‐Doped nano‐ZnO

**DOI:** 10.1111/1751-7915.13891

**Published:** 2021-10-22

**Authors:** Yuanzhe Li, Yang Liu, Bingqing Yao, Srikanth Narasimalu, ZhiLi Dong

**Affiliations:** ^1^ School of Materials Science & Engineering Nanyang Technological University Singapore 639798 Singapore; ^2^ School of Mechanical Engineering & Key Laboratory of Materials Design and Preparation Technology of Hunan Province Xiangtan University Xiangtan 411105 China; ^3^ Energy Research Institute @ NTU (ERI@N) CleanTech One Singapore 637141 Singapore

## Abstract

The recent COVID‐19 virus has led to a rising interest in antimicrobial and antiviral coatings for frequently touched surfaces in public and healthcare settings. Such coatings may have the ability to kill a variety of microorganisms and bio‐structures and reduce the risk of virus transmission. This paper proposes an extremely rapid method to introduce rare‐earth doping nano‐ZnO in polyamines for the preparation of the anti‐microbial polyurea coatings. The nano‐ZnO is prepared by wet chemical method, and the RE‐doped nano‐ZnO was obtained by mixing nano ZnO and RE‐dopants with an appropriate amount of nitric acid. This rapidly fabricated polyurea coating can effectively reduce bacteria from enriching on the surface. Comparing with pure nano‐ZnO group, all the polyurea coatings with four different rare‐earth elements (La, Ce, Pr and Gd) doped nano‐ZnO. The La‐doped nano‐ZnO formula group indicates the highest bactericidal rate over 85% to *Escherichia coli* (*E*. *coli)* and *Pseudomonas aeruginosa* (*Pseudomonas*). Followed by Ce/ZnO, the bactericidal rate may still remain as high as 83% at room temperature after 25‐min UV‐exposure. It is believed that the RE‐doping process may greatly improve the photocatalytic response to UV light as well as environmental temperature due to its thermal catalytic enhancement. Through the surface characterizations and bioassays, the coatings have a durably high bactericidal rate even after repeated usage. As polyurea coating itself has high mechanical strength and adhesive force with most substrate materials without peel‐off found, this rapid preparation method will also provide good prospects in practical applications.

## Graphical abstract

Antibacterial coatings are rapidly prepared by introducing RE‐doped nano‐ZnO in polyurea, and such coatings have a bactericidal rate over 85% to *E. coli* and *Pseudomonas*. Nano‐ZnO will generate ROS by the photocatalytic reaction to disrupt the bacteria from the surface, while the RE‐dopants can enhance the bactericidal effect of these coatings in the dark by thermal effect.

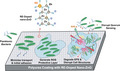

## Introduction

Studies have shown that the COVID‐19 virus can survive for up to 72 h on surfaces made of plastic and stainless steel. When the current global crisis has abated, efforts must turn to future preventative measures (Zuniga and Cortes, 2020). This virus has also led to a rising interest in antimicrobial coatings for frequently touched surfaces like door handles, shopping carts, elevator buttons and handrails, because the next people that come in there will re‐contaminate the surfaces even it was disinfected by the first one (Bukhari *et al*., 2020; Shetty *et al*., 2020). However, such antimicrobial coatings may have the ability to kill a variety of microorganisms and reduce the risk of virus transmission in most public and healthcare settings. It's not a substitute for regular cleaning and disinfecting, but an extra protective layer for the prevention of the bacteria and viruses (Taylor, 2020; Zhang, 2020).

The technology behind so‐called antimicrobial coatings has been around for almost a decade and has previously been used in hospitals to fight against the spread of infection, including against antibiotic‐resistant bacteria (Moore *et al*., 2013). There are numerous biological mechanisms to inhibit or eliminate bacteria formation on the surface, which include non‐specific inhibition (e.g. anti‐adhesive polymers), specific inhibition, disrupting signalling pathways, enzymatic action on EPS matrix and destruction of persisters (Driffield *et al*., 2008). Generally, the inorganic antimicrobial coating could make use of the slow exudation of toxic agents in the coating surface to form a toxic surface layer and kill most of the bacteria (Driffield *et al*., 2008). The metal silver is commonly used as an anti‐biofilm agent by depositing silver on the surfaces of biomaterials using coating technology (Yan and Bonnie, 2019). DNA was condensed and lost ability to replicate. Silver ion could also inactivate proteins by reacting with the thiol groups in cysteine residues. Because silver nanoparticles have an extremely large surface area, they can interact with microorganisms better. The nanoparticles could penetrate inside the bacteria, react with proteins and DNA, and interrupt the respiratory chain and cell division, leading to cell death (Yan and Bonnie, 2019). Recent research of Yeung et al. from Hong Kong University of Science and Technology also developed such an antimicrobial coating that is effective against COVID‐19 (Pemmada *et al*., 2020). It consists of millions of nano‐capsules containing low concentration silver‐based disinfectant which remain active for 90 days and was made available for commercial use this year following clinical tests at Hong Kong hospitals. In addition, the organic antimicrobial/ bactericidal coating, such as halogenated furanones, which is produced by red alga Delisea pulchra, can inhibit fouling of their surface (Gram *et al*., 1996; Dworjanyn *et al*., 2006). Furanones have been studied as a new class of anti‐microbial agents. Furanone was also covalently bonded to Silastic Tenckhoff catheters and rendered an inhibitory effect on biofilm formation (Cattò *et al*., 2018). Furthermore, in a sheep catheter infection model, furanone‐coated catheters tended to cause less severe infection than control catheters. Covalently coupled 3‐(trimethoxysilyl)‐propyl dimethyl octadecyl ammonium chloride (QAS) to silicone rubber will generate quaternary ammonium groups on the surface with antimicrobial activity. Quaternary ammonium functionalized silica nanoparticles were used to coat glass surfaces and exhibited inhibition of growth and accumulation of *gram*‐negative and *gram*‐positive bacteria on the surface. One of the obvious shortcomings of the bactericidal surfaces is that they could be covered by macromolecules and dead microorganisms and then lose their antimicrobial function (Bridier *et al*., 2011; Mah, 2012).

From the current trend of antibacterial paint development, the use of hydrophobic surface combined with antibacterial agents is a promising antibacterial means (Li *et al*., 2019b, 2020a). These mechanisms might include photocatalytic and/or EPS degradation, hydrophilic/hydrophobic surface structure and even quorum sensing. Recently, two‐component elastomeric polyurea (PUA) has received attention as a protective surface coating due to its unique characteristics such as fast setting, solvent‐free, resistant to a broad range of corrosives and solvents, excellent thermomechanical properties, adhesion and anti‐abrasive properties (Li *et al*., 2019b). In previous research, a new Titania‐polyurea spray antibacterial coating has also been fabricated, and such coating is able to prevent biofilm and bacteria from enriching on the surface and reduce the growth of mould effectively. It can utilize oxygen radicals generated to inhibit the growth of various microorganisms, or to destroy the cell structures of the microorganisms (Li *et al*., 2020a). Moreover, one similar photocatalyst, nano zinc oxide (nano‐ZnO), is of great research value for the antibacterial usage (Nagajyothi *et al*., 2014). Research led by Gouda *et al*. also explored the antiviral effect of nano‐ZnO mediated by hesperidin and in silico comparison study between antiviral phenolics, which may have potential treatment against SARS‐CoV‐2 virus (Attia *et al*., 2021). Mohamed *et al*. (2021) also investigated the Optimized Nanoscale Zinc Oxide against COVID‐19 through Antiviral Computational Analysis, which suggests the promising competence of the described ZnO nanoparticles for respiratory tract infection outbreaks. The introduction of a small amount of rare‐earth (RE) elements to nano‐ZnO can significantly improve its response to visible light and enhance its photocatalytic activity by prolonging the recombination time in between electron and hole (Jan *et al*., 2014). The rare‐earth (RE) element may absorb and emit electromagnetic wave radiation of various wavelengths from the ultraviolet region, visible region to infrared region (Deng *et al*., 2015). In this paper, the antimicrobial polyurea coatings with RE‐doped nano‐ZnO are obtained under a rapid preparation process. The antibacterial activities under different irradiation time, environmental temperature and light exposure, and antimicrobial durability have also been studied.

## Results and discussion

### Characterization and properties of polyurea coatings with RE‐Doped nano‐ZnO

#### X‐ray diffraction analysis

**Fig. 1:**
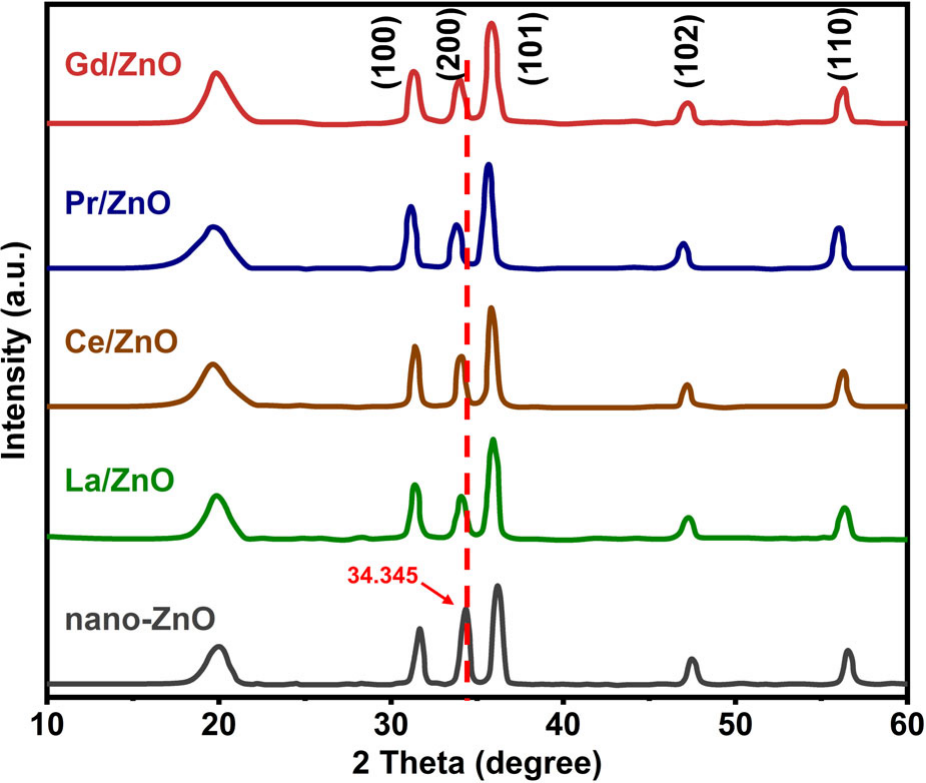
XRD patterns of polyurea coatings with (a) nano‐ZnO, (B) La/ZnO, (C) Ce/ZnO, (D) Pr/ZnO and (E) Gd/ZnO.

As X‐ray diffraction (XRD) could provide a more quantified result of the chemical composition of new fabricated coatings than energy dispersive X‐ray spectrometry (EDX) with qualitative analysis only, XRD analysis was finally selected to perform structural and crystal‐phase characterizations of the polyurea coating with nano‐ZnO and RE‐doped nano‐ZnO. From the results shown in Fig. 1, specific absorption bands of pure polyurea were not visible within the figure, and the diffraction peaks of RE‐doped ZnO polyurea coating samples were in accordance with the standard sample of hexagonal nano‐ZnO from the literature (Jan *et al*., 2014; Deng *et al*., 2015; Li *et al*., 2019b). Refined structural parameters of hexagonal nano‐ZnO at 300K are as follows: the lattice constants and unit cell volume are a = 3.245 Å, c = 5.2061 Å and V = 47.574 Å^3^ respectively. No impurity peaks were observed, such as La (La^3+^) cluster or La_2_O_3_, Ce (Ce^3+^) cluster or Ce_2_O_3_, Pr (Pr^3+^ & Pr^4+^) cluster or Pr_6_O_11_, Gadolinium (Gd^3+^) or Gd_2_O_3_, indicating that the RE‐dopants quantities were less than the solubility limits. The diffraction peaks of those four RE‐doped nano‐ZnO at 2θ have shifted towards lower angles compared with the nano‐ZnO (2 0 0) at 2θ = 34.345 °, which was due to the larger radius of RE‐dopants (Gd^3+^ = 0.93 Å, Pr^3+^ & Pr^4+^ = 1.12 Å & 0.96 Å, La^3+^ = 0.96 Å, Ce^3+^ = 1.02 Å) than that of Zn (0.74 Å) from top to bottom in Fig. 1. The peaks for polyurea coating with nano‐ZnO and RE‐Doped nano‐ZnO groups had slight difference from each other, indicating that the crystallographic positions of RE‐dopants ions have been successfully occupied in the ZnO host lattice and strain developed in the lattice. Besides, the alignment of polyurea chain remained regular and uniform even after the introduction of the nano‐ZnO and RE‐doped nano‐ZnO, which could guarantee good adhesion force in between those nanoparticles and the polyurea coating systems. It was also noted that polyurea coating with RE‐doped nano‐ZnO indicated a shift towards lower the value compared with the coating with pure nano‐ZnO nanoparticles due to the incorporation of RE‐metals into ZnO. And there were no other peaks of impurities detected in the XRD patterns of these coating samples.

#### Morphological characterization

**Fig. 2:**
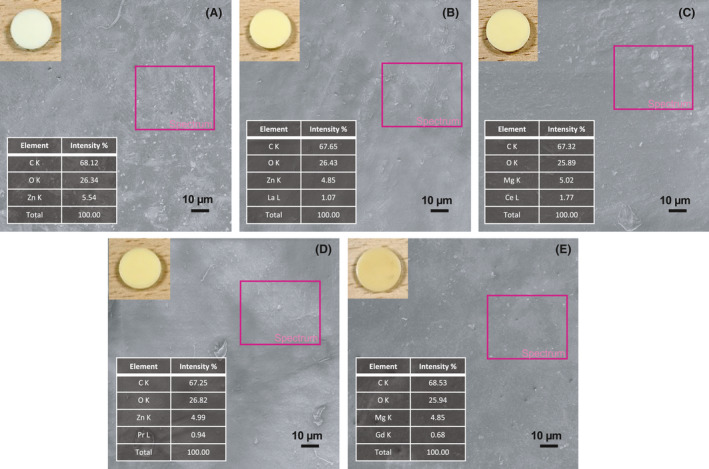
SEM micrographs of polyurea coatings with (A) nano‐ZnO, (B) La/ZnO, (C) Ce/ZnO, (D) Pr/ZnO and (E) Gd/ZnO.

Scanning electron microscopy (SEM) imaging in Fig. 2 exhibited the surface morphology of polyurea coatings with (a) pure nano‐ZnO, and (b–e) RE‐doped nano‐ZnO respectively. Most of the coating samples would exhibit similarly punctiform surface morphology. With the exception of Pr/ZnO coating sample, the other rapidly fabricated polyurea coatings all illustrated micro‐scale convex structures with 2.0–10.0 µm of diameters randomly oriented and aggregated on their surfaces, which supposed to be the platelets of the nano‐ZnO or RE‐doped nano‐ZnO (Jan *et al*., 2014; Deng *et al*., 2015). The samples with Pr/ZnO as shown in Fig. 2E exhibited the mixed structures of both platelets and rods with flat surface morphology, which might be caused by the internal forces between polyurea and the Pr/ZnO nanoparticles.

**Fig. 3:**
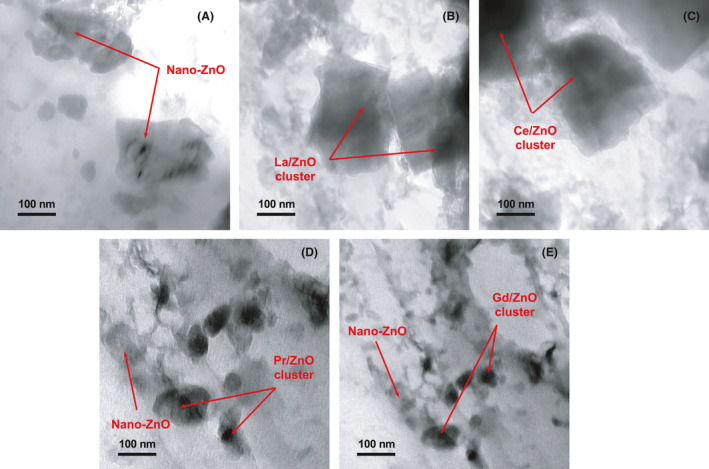
TEM micrographs of polyurea coatings with (A) nano‐ZnO, (B) La/ZnO, (C) Ce/ZnO, (D) Pr/ZnO and (E) Gd/ZnO.

Figure 3 depicted the correspondent transmission electron microscopy (TEM) imaging of polyurea coatings with (a) pure nano‐ZnO, and (b–e) RE‐doped nano‐ZnO as SEM respectively. In the cases of Lanthanum (La) and Cerium (Ce) doped nano‐ZnO (Fig. 3B and C), polyurea coating was able to observe the ZnO platelet with hexagonal or similar crystal shape clearly. This observation was in agreement with the TEM imaging of Cerium and Lanthanum oxides (CeO_2_ and La_2_O_3_) diffraction peaks during the synthesis process of RE‐Doped nano‐ZnO (Li *et al*., 2014, 2017; Lahmer, 2018). And the rare elements were able to be observed in energy dispersive spectrometer (EDS) mappings as well. However, all the TEM micrographs for these polyurea coatings with RE‐doped nano‐ZnO still revealed similar micro‐structures as the ones with nano‐ZnO. It needed to be noted that, as the coating samples were not fully transparent, it might be not possible to see evidence of other phases of nano‐ZnO or RE‐doped nano‐ZnO other than the shadows which could be related to both dispersion of the RE‐Doped nano‐ZnO particles and/or the structural polyurea coating itself (Kaneva *et al*., 2015). The imaging of Gd/ZnO groups in Fig. 3E also indicated the smallest crystal structures of Gd doped nano‐ZnO among all the polyurea coating groups, which could be correlated to the monoclinic or cubic crystal structure of the gadolinium oxide (Gd_2_O_3_).

#### UV‐Vis diffuse reflectance spectra analysis

**Fig. 4:**
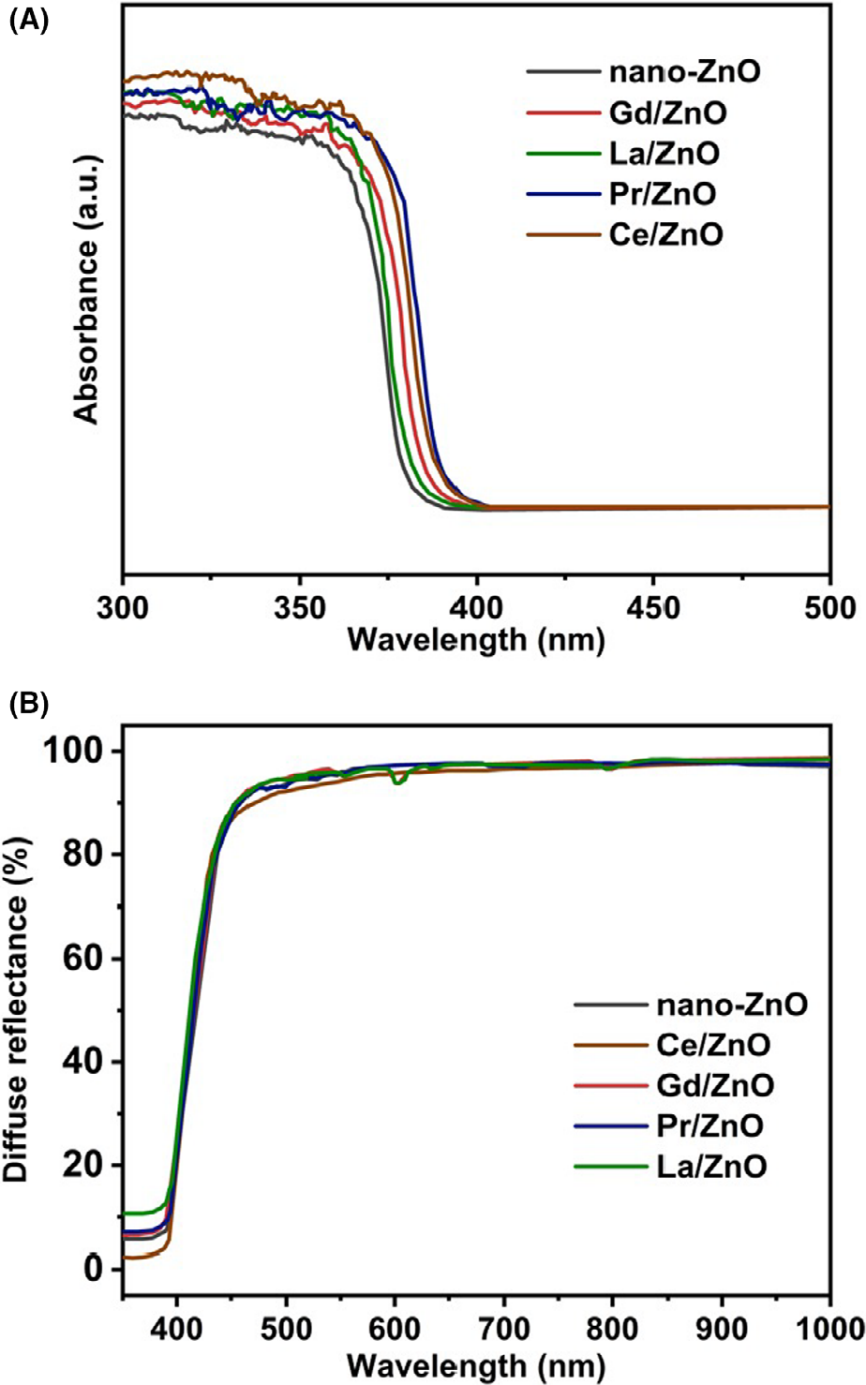
(A) UV‐Vis absorption, and (B) diffuse reflectance spectra of polyurea with nano‐ZnO, La/ZnO, Ce/ZnO, Pr/ZnO and Gd/ZnO.

The introduction of pure nano‐ZnO and RE‐doped nano‐ZnO in polyurea coatings played an important role in the optical properties and bactericidal activities. The absorption and reflectance spectrums of the various coatings were investigated with diffuse reflectance (DR) UV‐visible (UV‐Vis) spectroscopy. The absorption spectrum of polyurea with nano‐ZnO as well as RE‐doped nano‐ZnO was indicated in Fig. 4A. The spectra of the coating with nano‐ZnO only exhibited a strong absorption band at about 329 nm (Lahmer, 2018). Due to the band‐gap transition from the valence band (VB) to the conduction band (CB) of the pure nano‐ZnO, the coating samples with nano‐ZnO had the same band‐gap transition at about 386 nm (3.29 eV). However, the use of different RE‐doped nano‐ZnO dopants did not change the absorption band of the rapidly fabricated polyurea coatings. The values for all RE‐doped samples closely approximated to the nano‐ZnO group, indicating that the RE doping in the nano‐ZnO matrix did not affect directly the transition of zinc oxide (Li *et al*., 2014, 2017; Deng *et al*., 2015). In the case of coating with Cerium (Ce) doped nano‐ZnO, it was able to detect a slightly wide wavelength range as well as high absorbance even at the same wavelength after comparing with other coating groups, which demonstrated its outstanding optical properties (Li *et al*., 2014, 2017; Lahmer, 2018). This was because, on the one hand, Cerium (Ce), as a rare earth element, has a complex energy level structure, and the doping of Ce^3+^ ions into nano‐ZnO would introduce new impurity energy levels in its forbidden band, reduce the forbidden band width, lower the band gap energy and expand the response range of the ZnO absorption spectrum. Thus, it further improved the photon utilization and enhanced the photocatalytic activity of nano‐ZnO. On the other hand, the presence of unpaired 4f layer electrons in the electronic layer structure of Ce (4f^1^ 5d^1^ 6s^2^) could promote interfacial charge transfer and inhibit the complexation of photogenerated electron–hole pairs. Ce^3+^ ions, as a Lewis acid, could trap electrons more easily than O_2_ molecules. And when nano‐ZnO was photoexcited and generated electrons, the Ce^3+^ at the interstitial position could effectively trap them and make them react with the O_2_ molecules to generate superoxide ion radicals (⋅O^‐2^). However, when the doping of Ce^3+^ was too much, it would again become the compound centre of the electron–hole pair, accelerating the electron–hole compound, but inhibiting the photocatalytic performance of nano‐ZnO. Finally, for all the RE‐doped nano‐ZnO coatings, they all contained a small overlap of absorption extending with the visible region, which provided the indirect evidence for the La/ZnO and Ce/ZnO polyurea coating sample obtaining the outstanding bactericidal activities in the following sections.

The UV‐Vis DRS in Fig. 4B illustrated the corresponded reflectance spectra of polyurea with nano‐ZnO and RE‐doped nano‐ZnO with band edge between 355 and 1000 nm. It showed that the band gap of ZnO was less affected by the doping of different rare earth elements, and the absorption band edge was unchanged or slightly blue‐shifted when compared with undoped nano‐ZnO. The diffuse reflectance spectrum exhibited a slight fluctuation between 900‐1,000nm. This might be due to the presence of the intermediate states of RE‐dopants and nano‐ZnO in the band gaps (Kaneva *et al*., 2015; Pascariu *et al*., 2019). Last but not the least, all polyurea coating samples indicated a high diffuse reflectance percentage at 95% above.

### Antibacterial activity

*Escherichia coli* and *Pseudomonas aeruginosa* are two types of bacteria that commonly used in the laboratory (Li *et al*., 2019b, 2020a,b). Both bacteria can be found on many frequently touched surfaces and may be selected as representatives in the bacteria growth and dispersal. All the *P*‐values for the following statistical significances were <0.05.

#### Effect of irradiation time on antibacterial activity

**Fig. 5:**
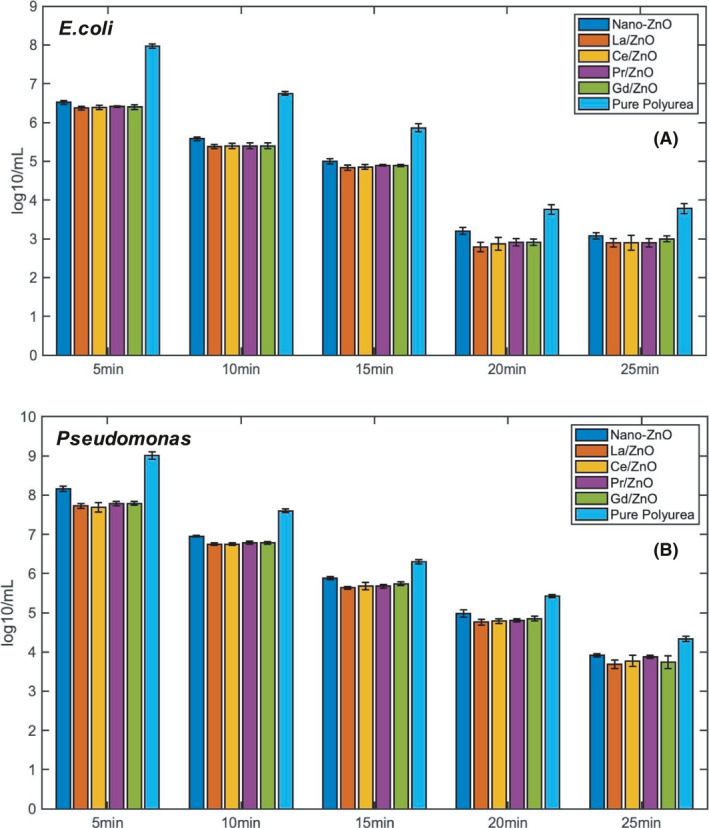
Increment of antibacterial effect with longer time intervals within (A) *E. coli* CDC‐reactor and (B) *Pseudomonas* CDC‐reactor.

Typically, the biofilm attachment, formation and detachment began with (i) the transport and initial adhesion of the planktonic bacteria through the adsorption of suspended particles and organic species from the bulk fluid, (ii) transport and attachment of the planktonic cells, (iii) microbial multiplication and EPS production and (iv) dispersal and detachment of the mature clusters (Iqbal *et al*., 2009; Pascariu *et al*., 2019; Li *et al*. 2019a). Under the irradiation of the UV light, reactive oxygen species (ROS) as illustrated in Fig. S1 would generate by the nano‐ZnO, which could damage of DNA of the biofilm, oxidize the fatty acids in lipid, and result in significant damage to cell structures (Li *et al*., 2019a, 2020a). The antibacterial effect of the rapidly fabricated polyurea coatings against *E. coli* and *Pseudomona* increased significantly with the increment of the irradiation time when UV light was applied (Fig. 5) as more ROS would be generated after a longer time of UV‐exposure. For polyurea coating with nano‐ZnO only, the amount of viable *E. coli* cell (Fig. 5A) decreased from 6.52 log ml^‐1^ at 5 min to 3.20 log ml^‐1^ at 25 min, which was already considered a low attachment rate. Compared with *E. coli* CDC reactor, the colony forming unit (CFU) count of the *Pseudomonas* was almost ten times more. However, similar decreasing scenarios happened within the *Pseudomonas* CDC‐reactor as indicated in Fig. 5B. The amount of viable *Pseudomonas* cell decreased from 8.16 log ml^‐1^ at 5 min to 3.92 log ml^‐1^ at 25 min. However, in the control groups of pure polyurea coating groups, which were not exhibited in Fig. 5, the antibacterial effect would also increase with the irradiation time. It was because the UV light could also degrade the biofilm in the absence of any nano‐ZnO.

Moreover, after the introduction of RE‐doped nano‐ZnO, specially for La/ZnO and Ce/ZnO groups, the bactericidal effects increased greatly with the irradiation time, which was supposed to be caused by the high photocatalytic performance of Lanthanum (La) and Cerium (Ce) dopants as the previous UV‐Vis diffuse reflectance spectra analysis. In the *E. coli* CDC‐reactor, the CFU count of both La/ZnO and Ce/ZnO coating groups would decrease from 6.40 log ml^‐1^at 5 min to 2.79 log ml^‐1^ log ml^‐1^ at 25 min, which indicated sufficient evidence of the rapidly fabricated polyurea coatings was able to reduce the attachment of the biofilm at the early stage. While for other two RE‐doped nano‐ZnO coating groups, Pr/ZnO and Gd/ZnO, though the efficiency was not as high as previous two groups, they were just right behind (2.79–2.87 log ml^‐1^ at 25 min for both groups) (Iqbal *et al*., 2009). In the *Pseudomonas* CDC‐reactor, La/ZnO and Ce/ZnO coating groups still took the lead, and the CFU count decreased from 7.78 log ml^‐1^ at 5 min to 3.70 log ml^‐1^ at 25 min, which shared the same trend as *E. coli* group of testing. However, this time the Gd/ZnO exceeded Pr/ZnO, and the final CFU count was even slightly lower than Ce/ZnO with 3.74 log ml^‐1s^ at 25 min.

#### Effect of environmental temperature on antibacterial activity

**Fig. 6:**
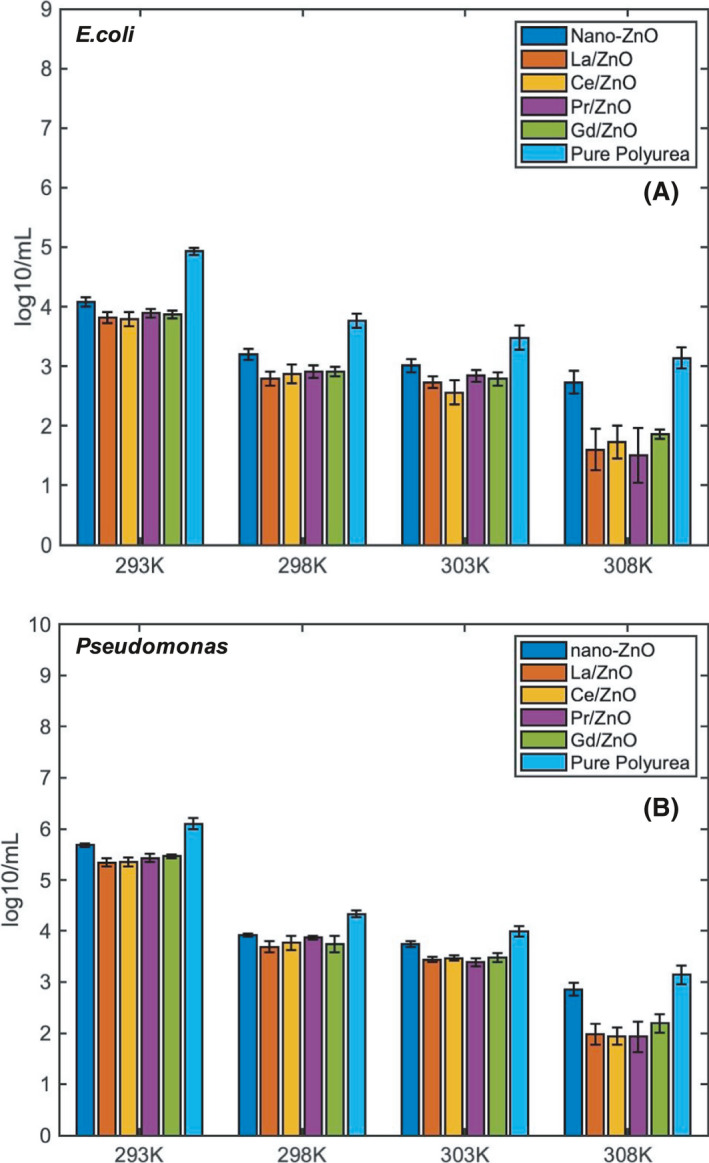
Increment of antibacterial effect with higher environmental temperatures within (A) *E. coli* CDC‐reactor and (B) *Pseudomonas* CDC‐reactor.

As indicated in the Fig. 6, it could be confirmed that all these rapidly fabricated coatings could have greater *E. coli* removal effect at high environmental temperature. In the *E. coli* CDC‐reactor, the CFU count of *E. coli* cells for nano‐ZnO group decreased from 4.08 log/mL at 293 K to 2.73 log/mL at 308 K, other four RE‐doped nano‐ZnO coating groups illustrated a greater reduction from 3.79–3.87 log ml^‐1^ at 293 K to 1.51–1.81 log ml^‐1^ at 308 K. In the *Pseudomonas* CDC‐reactor, the CFU count of *E*. *Pseudomonas* cells for nano‐ZnO group decreased from 8.16 log ml^‐1^ at 293 K to 3.92 log ml^‐1^ at 308 K, other four RE‐doped nano‐ZnO coating groups illustrated a greater reduction from 7.69–7.78 log ml^‐1^ at 293 K to 3.69–3.87 log ml^‐1^ at 308 K.

These results indicated that, except for the photocatalytic activity triggered by UV light only, the thermal effect of the RE‐doped nano‐ZnO would also contribute to high concentration of generated ROS and the reduction in attached *E. coli* and *Pseudomonas* (Li *et al*., 2019a). Moreover, it was interesting to note that at 308 K, the amount deviation of viable cell varied in a larger range than at low temperature, which should be closely related to the active degree of the *E. coli* cells and the temperature preference of *E. coli* clusters growth. And for both *E. coli* and *Pseudomonas* groups, there was a significant drop of CFU count between 303 and 308 K, which was almost half the CFU count for these RE‐doped nano‐ZnO formula groups. It was believed that, at high‐temperature conditions, the planktonic bacteria would increase its transport and quorum sensing/quench between the polyurea coating surface. Besides, the quorum sensing/quenching effect would also disturb the communication between the bacteria cells by causing much signal molecule degradation and signal reception disruption (Flemming and Wingender, 2010). Due to the higher temperature than 308 K, as recorded in the recent publications, also might cause the death of biofilm, so the temperature was kept within 308 K only. And it was believed that if it kept increasing to 313 K and 323 K, there would be more significant decrease in biofilm due to the thermal catalytic effect of RE (Farha and Brown, 2013; Kiymaci *et al*., 2018).

#### Effect of light exposure on antibacterial activity

**Fig. 7:**
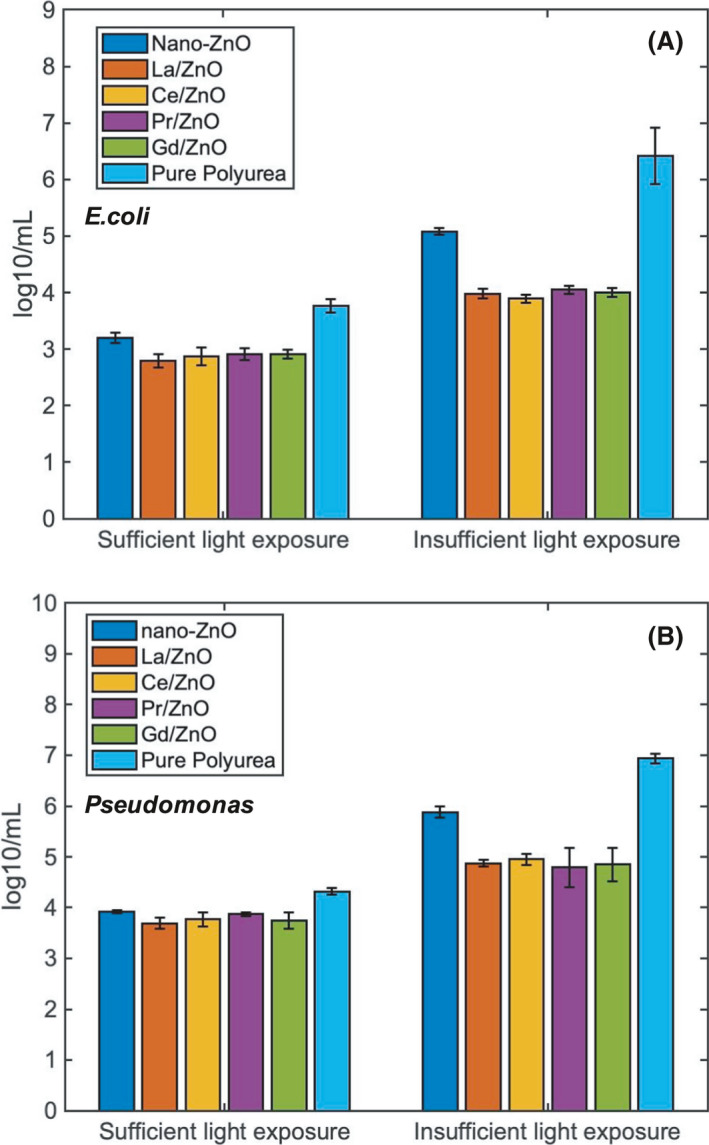
Comparisons of antibacterial activity under different light exposure conditions within (A) *E. coli* CDC‐reactor and (B) *Pseudomonas* CDC‐reactor.

The antibacterial activity under different light exposure was indicated in Fig. 7. The result was obtained at 298 K after 25 min treatment. The polyurea coatings under sufficient light exposure would always indicate better bactericidal effect than the insufficient ones for both bacteria CDC‐reactor, which indicated the reliability of the photocatalytic activities under UV irradiation and the high concentration of generated ROS. And the four coating groups with RE‐doped nano‐ZnO illustrated lower the CFU count of viable bacteria cells than the coating group with nano‐ZnO only, which indicated the accumulated thermal‐enhancement effect caused by the RE‐dopants (Farha and Brown, 2013). The La/ZnO and Ce/ZnO coating groups were still the ones with the best performance among all the five rapidly fabricated polyurea.

At insufficient conditions, as indicated on the right side of Fig. 7, the growth rate of *E. coli* was a bit higher than the *Pseudomonas*. Compared with the sufficient light conditions, there was a significant increase in CFU count for nano‐ZnO group, as the photocatalytic effect was not triggered, and the rough surface might create the spatial structures for the attachment of more *E. coli* and *Pseudomonas* cells. The amount of viable bacteria cells could still remain at 5.08 log/mL for *E. coli* (Fig. 7A) and 5.88 log/mL for *Pseudomonas* (Fig. 7B). While for the Re‐doped nano‐ZnO coating groups, the antibacterial effect was still close to the sufficient ones, which was supposed to be contributed by the RE as discussed in the previous section.

### LIVE/DEAD Biofilm imaging

**Fig. 8:**
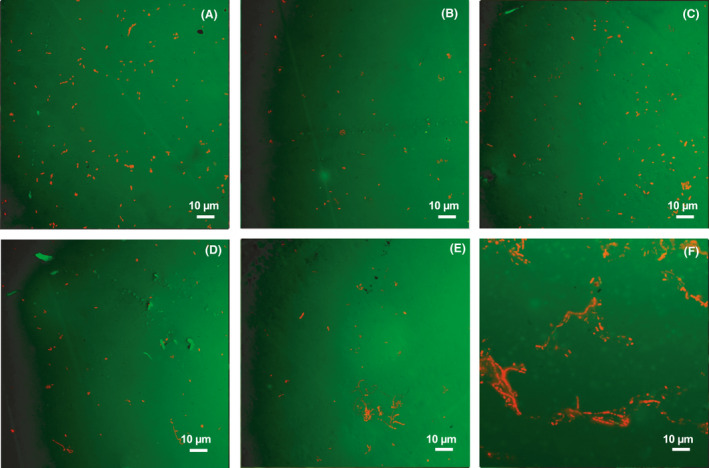
LIVE/DEAD Biofilm imaging of polyurea coatings with (A) nano‐ZnO, (B) La/ZnO, (C) Ce/ZnO, (D) Pr/ZnO, (E) Gd/ZnO and (F) pure polyurea in *E.coli* CDC‐reactor.

**Fig. 9:**
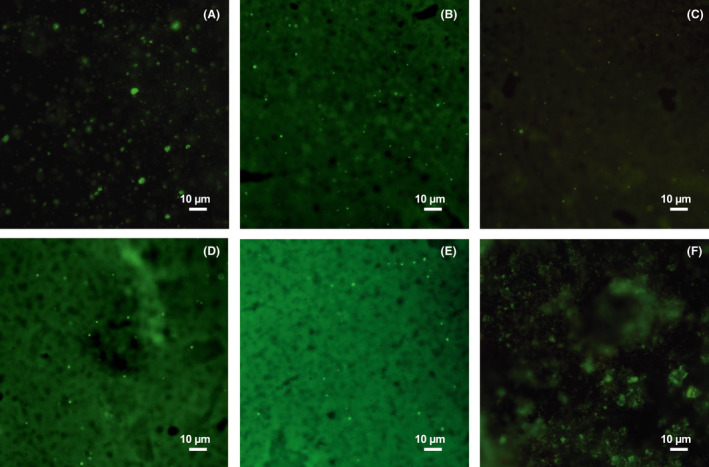
LIVE/DEAD Biofilm imaging of polyurea coatings with (A) nano‐ZnO, (B) La/ZnO, (C) Ce/ZnO, (D) Pr/ZnO, (EE) Gd/ZnO and (F) pure polyurea in *Pseudomonas* CDC‐reactor.

Comparable runs of the biofilm LIVE/DEAD stain on the *E. coli and Pseudomonas* biofilms grown on the coupon and duplicate images of the five different coupons under conditions were performed. Figures 8 and 9 indicated all six most optimized polyurea coating groups under at 308 K after 25 min treatment. Both LIVE and DEAD imaging of bacteria cells were captured from these coupons. Cells with compromised membranes that were considered to be DEAD or dying would stain brightly red, whereas the LIVE cells with an intact membrane would stain brightly green. However, as the coupon had a strong autofluorescence signal from the Syto9 wavelength, so some signals from LIVE cells might be brighter in the biofilm imaging of *Pseudomonas* (Li *et al*., 2019b).

In Fig. 8, barely visible LIVE *E. coli* cells could be observed on those rapidly fabricated polyurea coating surfaces, which illustrated the low protein adsorption and biofilm adhesion as well as the unique bactericidal effect. The flat coating surface could also increase the difficulties of the initial adhesion for the planktonic *E. coli* cells. Particularly, there was some difference among the comparisons in the DEAD biofilm imaging. The polyurea coating group with nano‐ZnO in Fig. 8A had more stained red dye than any other coating group (Fig. 8B–E) with RE‐doped nano‐ZnO, which illustrated a higher initial adhesion of the *E. coli* cells. The elimination of the *E. coli* cells would highly depend on the near‐wall photocatalytic effect/photodegradation for the nano‐ZnO group. While for other four RE‐doped nano‐ZnO groups, the dead cells would only accumulate on the rough or concave portion of the coating surface, which was caused by the fluid convection factors. And there would be higher concentration of near‐wall ROS than nano‐ZnO groups, which enabled the formation of a protective layer to prevent the *E. coli* cells from adhering or enriching on the surface (Farha and Brown, 2013; Li *et al*., 2021). In Fig. 8F, the stained red structure illustrated the high concentration of *E. coli* cells, which was caused by the quick growth and dispersal. In Fig. 9A–F, LIVE *Pseudomonas* could only be observed at Nano‐ZnO and pure polyurea coating samples with stained green signage. The four RE‐doped still remained clear after the same exposure as *E. coli*, which clearly proved their antibacterial effect for both bacteria cells. The DEAD biofilm imaging was not obvious for *Pseudomonas* as strong autofluorescence happened. Moreover, a simple leaching test for the liquid, through nano‐particle detection, within the fresh and waste carboys was conducted every time the coupons were put and withdrawn from the CDC reactor (Li *et al*., 2019b, 2020b). The testing method was the same as the Zeta Potential test. No significant difference between the fresh and waste carboy was detected, which could be considered as limited impact of RE‐doped nano ZnO on the environment and safety for human use. Still, whether the nanoparticles, nano‐ZnO or RE‐doped nano‐ZnO, would contribute to the antibacterial effect was under further investigation.

### Antimicrobial durability characterization

The antimicrobial durability of these rapidly fabricated polyurea coatings is one of the key indicators for sustainable use. From the experimental results illustrated in Tables 1 and 2, the La/ZnO group remained good antibacterial properties of both *E. coli* at 83.9% and *Pseudomonas* at 82.9% even after continuous use for five times. Followed by Ce/ZnO group, it indicated the second highest antimicrobial efficiency at 79.6% for *E. coli* and 80.8% for *Pseudomonas*. Even after repeated washing, drying, medium immersion, etc., the coating surface film layer still indicated a significant antibacterial effect. The surface of all other polyurea coatings was also flat and clean, and there was no signage of scratches or cuts, which was contributed by the high mechanical strength and the self‐healing effect of polyurea (Li *et al*., 2019b).

**Table 1:** Antimicrobial durability (*E. coli*) of polyurea coatings with RE‐Doped nano‐ZnO.

	Nano‐ZnO	La/ZnO	Ce/ZnO	Pr/ZnO	Gd/ZnO
1	43.2 ± 1.3	85.5 ± 0.7	83.4 ± 1.9	79.5 ± 2.6	78.9 ± 1.4
2	38.3 ± 2.9	85.8 ± 2.2	81.4 ± 2.9	76.9 ± 1.4	79.5 ± 2.1
3	40.3 ± 1.5	85.1 ± 1.7	80.4 ± 1.8	74.5 ± 2.2	74.6 ± 1.7
4	38.7 ± 2.7	84.4 ± 1.9	79.4 ± 1.4	76.8 ± 2.1	77.7 ± 2.3
5	37.5 ± 1.6	83.9 ± 3.7	79.6 ± 2.1	74.9 ± 2.9	76.5 ± 1.4

**Table 2:** Antimicrobial durability (*Pseudomonas*) of polyurea coatings with RE‐Doped nano‐ZnO.

	Nano‐ZnO	La/ZnO	Ce/ZnO	Pr/ZnO	Gd/ZnO
1	51.2 ± 2.3	83.9 ± 1.2	85.2 ± 1.2	81.0 ± 1.6	79.6 ± 2.4
2	47.5 ± 0.8	84.2 ± 0.8	83.3 ± 0.9	79.2 ± 2.1	78.4 ± 1.3
3	43.1 ± 2.1	82.3 ± 1.6	81.9 ± 2.3	78.9 ± 2.2	77.9 ± 2.1
4	39.7 ± 1.4	83.1 ± 1.1	80.4 ± 2.2	76.8 ± 1.8	74.8 ± 1.3
5	40.6 ± 1.2	82.9 ± 1.8	80.8 ± 1.3	76.9 ± 1.1	75.1 ± 2.4

To conclude, the antibacterial polyurea coating was able to be prepared within seconds, and such coating with RE‐doped nano‐ZnO, especially Ce/ZnO and La/ZnO, showed a significant reduction in both *E. coli* and *Pseudomonas* adhesion. Due to the colloidal stability and strong adsorption of the RE‐doped nano‐ZnO, the bacteria lost its living conditions and died (Iqbal *et al*., 2009; Li *et al*., 2020b; Attia *et al*., 2021). Besides, as a key step in bacteria growth, preventing planktonic bacteria cells entry into the host cell is a new strategy for antibacterial. Certain small molecules could bind to the conserved spatial structure of bacteria surface proteins, destroy the bacteria structures and even direct inactivation of those viruses. Furthermore, such nanomaterials could bind to viral nucleic acids and change the structure of viral DNA or RNA, affecting the replication of viral DNA or RNA and rendering the virus inactive (Imani *et al*., 2020; Mohamed *et al*., 2021). Specially at long time irradiation and high environmental temperature, the RE‐dopants inside the coating systems would also illustrate its thermal catalytic effect, which would trigger and enhance the photocatalytic reaction of the nano‐ZnO. Such enhancement character contributed by the RE‐dopants would guarantee the near‐wall ROS remaining at high concentration level. The ROS would destroy the cell membranes, interrupt the cell communication (quorum sensing) and even degrade the formation of EPS at the early stage of the adhesion. And such reduction or inhibition of any other biofilm should be non‐selective. Moreover, even after repeatable use of these coatings, the antibacterial activities were not dramatically declined.

Antibacterial and antiviral have been the mainstream technology for the future coating market. High‐quality nano‐ZnO might be used as the next generation of antibacterial and antiviral agent after silver nanoparticles. These rapid prepared polyurea coatings with RE‐doped nano‐ZnO could be used in medical context surfaces, door handles, shopping carts, elevator buttons and handrails to enhance their antibacterial and anti‐viral capabilities. Although both *E. coli* and *Pseudomonas* might prove the applicability and feasibility in the practical usage for such coatings, still more types of bacteria as well as virus were supposed to be tested and studied in the near future. The antiviral mechanism of such nano antibacterial material may be related to the mechanical adsorption and immobilization of *E. coli* by RE‐doped nano‐ZnO, as the surface could immobilize a large number of proteins and enzymes, especially polysaccharides, thus preventing the adsorption of viruses to host cells and showing strong antiviral activity (Farha and Brown, 2013; Li *et al*., 2019b; Mohamed *et al*., 2021). Although many antiviral studies of nano‐ZnO coating have been documented in recent search, the antiviral mechanism of such antibacterial coatings still needs to be further investigated.

## Conclusion

In summary, antibacterial coatings are well and rapidly prepared by introducing RE‐doped nano‐ZnO in polyurea in this study. These polyurea coatings with La, Ce, Pr and Gd (RE‐dopants) doped nano‐ZnO indicate a high bactericidal rate over 85% to *E. coli* and *Pseudomonas*. The nano‐ZnO in the coating system can generate ROS by photocatalytic reaction and prevent the bacteria cells from enriching on the surface at the early stage. While the RE‐dopants can enhance the bactericidal effect of the coatings in the dark by their thermal catalytic effect especially at high temperature. Moreover, these rapid prepared coatings also exhibit high antimicrobial durability after repeatable use. These rapidly prepared polyurea coatings with good antibacterial activities indicate great potential for the applications for medical constant and clinical devices, food packaging and fibre or frequently touched surfaces to prevent microbial infection and contamination.

## Experimental procedures

### Materials

Dragonshield‐BC^TM^ (Washington, DC, USA) 50–80 wt.% isocyanates (TDI) and 50–90 wt.% polyether amines (Polyoxypropylenediamine) were used to fabricate polyurea. Oligomeric diamines P1000, an alternative choice of polyether amines, was purchased from Versalink (Essen, Germany). Polydimethylsiloxane (PDMS) (average Mn ~ 2500), agent analytical reagent grade triethylamine (≥ 99.5%), zinc acetate dihydrate (≥ 98%), rare‐earth nitrate Cerium (II) nitrate hexahydrate (Ce(NO_3_)_3_・6 H_2_O), rare‐earth oxides Lanthanum (III) oxide (La_2_O_3_), Praseodymium (III,IV) oxide (Pr_6_O_11_), Gadolinium (III) oxide (Gd_2_O_3_) all under 99.99% trace metals basis, 99% diethanolamine purity, 99.5% glacial acetic acid, other reagents are analytical reagent grade, are from Sigma Aldrich (Bangkok, Thailand) as provided.

### Synthesis of RE‐Doped nano‐ZnO

The nano‐ZnO was prepared by wet chemical method. At first, zinc acetate dihydrate was dissolved in 50 ml of deionized water with stirring at room temperature. After the triethylamine was added in, stirring at 800 rpm was continued at room temperature for another 8 h followed by the literature (Li *et al*., 2014, 2017). The solution was followed by centrifugation, and the precipitate was washed with deionized water and absolute ethanol. Then, the rare‐earth oxides were weighed and dissolved together using appropriate amount of nitric acid with the precipitate at solution pH 7.4. Finally, the mixture solution was stirred at 85°C for another 0.5 h at 1200 rpm and oscillated by KQ‐400DB CNC ultrasonic cleaning machine (Orioner, KL, Malaysia). After drying under 60°C for 1.5 h, rare‐earth‐doped nano‐ZnO was obtained. The doping amount of rare‐earth element was 0.28 wt.%, 0.31 wt.%, 0.35 wt.% and 0.42 wt.% for La/ZnO, Ce/ZnO, Pr/ZnO, and Gd/ZnO respectively (Kaneva *et al*., 2015).

### Preparation of polyurea coatings with RE‐Doped nano‐ZnO

After conducting several pre‐experiments, 1.5 wt.% of RE‐Doped nano‐ZnO inside the coating system was proposed for the ideal preparation of polyurea coatings with RE‐doped nano‐ZnO, which were named as La/ZnO, Ce/ZnO, Pr/ZnO, and Gd/ZnO. Another two control groups of pure polyurea coating without nano‐ZnO and 1.5 wt.% undoped nano‐ZnO polyurea coatings were fabricated. Besides, 1.5 wt.% of polydimethylsiloxane (PDMS) were applied for all formula group as the deforming agent. Mixing of polyurea coating was carried out with vacuum level of 0.5 kPa to remove bubbles by Kakuhunter SK‐300TVSII mixer (Shashin Kagaku Pte Ltd, Shiga, Japan). For better mixture of these two main components, all chemicals were raised to 70 °C to decrease their viscosity before putting into the mixer for 180 s (Li *et al*., 2019b). The revolution speed and rotation speed were set at 580 rpm and 1700 rpm respectively. After mixing, the polyurea was poured into Teflon modes. The model was made in dimension of 100 × 100 × 10 mm. After pouring into the mode, polyurea was put into oven under 70°C for curing of 48 h and waiting for the following materials characterization.

### Characterization and properties of Polyurea Coatings

The X‐ray diffraction (XRD) measurements were performed by XRD‐600 (Shimadzu, Tokyo, Japan). The morphology of the materials was studied by scanning electron microscopy (SEM) using a JEOL microscope (JSM‐6510‐LV; JEOL, Tokyo, Japan) and by a transmission electron microscopy (TEM) using a JEOL JEM 3010 (300 kV) microscope. High‐resolution spectra were collected using 25 eV at 0.1 eV steps with a chamber pressure below 7.5 × 10^−9^ mbar. Five images were taken for each surface at a working distance of 7 mm and a potential of 5 kV. The UV‐Vis absorption spectra were recorded using a Varian Cary 5000 spectrophotometer, coupled with an integration sphere for diffuse reflectance studies (DRS), using a Carywin‐UV/scan software (Li *et al*., 2019b, 2020a).

### Measurement of antibacterial activities

For the antimicrobial evaluations, colonies of *Escherichia coli* (*E. coli*, gram‐negative, 8099) and *Pseudomonas aeruginosa* isolated from the local drinking water distribution system were used for antibacterial activity assessment by CDC biofilm reactor (BioSurface Technologies Corp., Bozeman, MT, USA). The bacterium was cultured in a nutrient‐rich distilled water medium under the ambient temperature of an average 25°C, 105 kPa for 20 min. Cultures in the nutrient media were diluted to 0.05 OD600 in distilled water and then cultured for 24 h under ambient temperature, at 150 rpm stirring speed, with a batch reactor setup. The source of UV‐irradiation remained the same as previous bioassays (Li *et al*., 2019b, 2020a). Coupons were collected after 5, 10, 15, 20 and 25 min of exposure, and comparison in between different environmental temperature (20, 25, 30 and 35°C) and different light exposure conditions with one half‐shaded from UV irradiation (Insufficient condition) and another exposed to ambient light (Sufficient condition) were performed. The biofilms on the smooth side of the coating were stained with the FilmTracer LIVE/DEAD Biofilm Viability Kit (Thermo Fisher Scientific, Waltham, MA, USA). Imaging was performed using the Carl Zeiss LSM 780 laser scanning confocal microscope (Zeiss, Jena, Germany). For the durability testing of the newly fabricated polyurea coatings, all polyurea samples were tested under 25°C and sufficient UV condition with 25 min exposure after disinfection from the previous test (Li *et al*., 2020a).

## Funding Information

This research was funded by MOE Academic Research Fund (AcRF) Tier 1 Project ‘Nano‐structured Titania with tunable hydrophilic/hydrophobic behaviour and photocatalytic function for marine structure application’, Grant Call (Call 1/2018) _MSE (EP Code EP5P, Project ID 122018‐T1‐001‐077), Ministry of Education (MOE), Singapore.

## Conflicts of interest

The authors declare no conflict of interest.

## Supporting information


**Fig. S1**. Scheme of bacterial deactivation by the free radicals generated using RE‐ZnO under UV and visible light.
