# Supplementary figures and images for: Rapid preparation and antimicrobial activity of polyurea coatings with RE‐Doped nano‐ZnO
Source: Microb Biotechnol. 2021 Oct 22;15(2):548–60. doi: 10.1111/1751-7915.13891 (PMC8867993; doi:10.1111/1751-7915.13891)

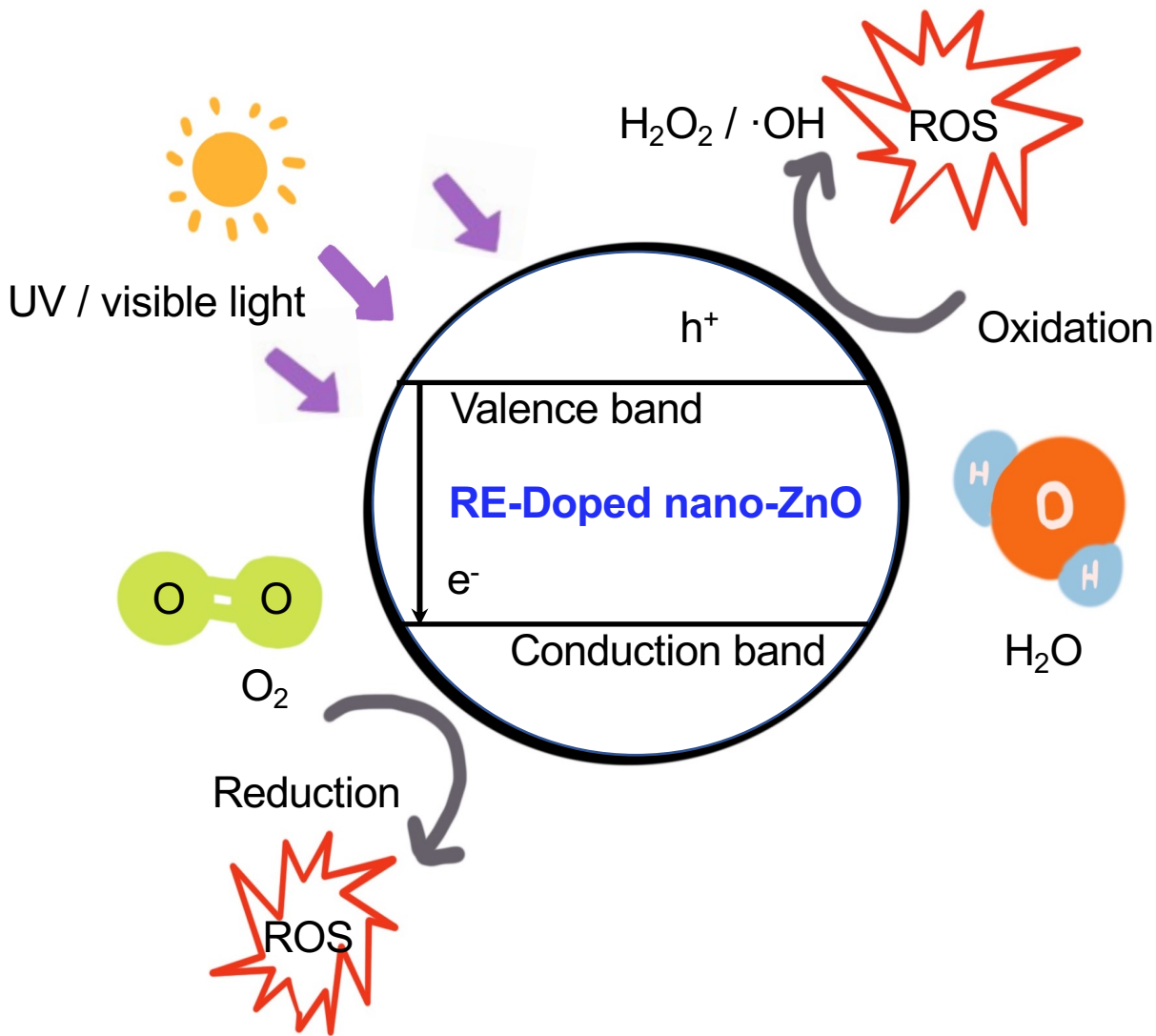

Supplement: Supplementary file 1 — Fig. S1. Scheme of bacterial deactivation by the free radicals generated using RE‐ZnO under UV and visible light. [file MBT2-15-548-s001.pdf]
